# How much is too much?: A retrospective causal analysis of the 7-day fluid balance for septic critical care patients

**DOI:** 10.1097/MD.0000000000040733

**Published:** 2024-12-27

**Authors:** Zheng Yang, Zhanli Shi, Wenwen Song

**Affiliations:** a Department of Intensive Care Unit, Hangzhou Red Cross Hospital, Hangzhou, China; b Department of Radiology, The First Affiliated Hospital of Zhejiang Chinese Medical University (Zhejiang Provincial Hospital of Chinese Medicine), Hangzhou, China.

**Keywords:** fluid balance, intravenous fluid, mortality, restrictive fluid, resuscitation, sepsis, septic

## Abstract

Many studies have provided significant evidence to suggest that early aggressive fluid resuscitation strategies are acutely beneficial in patients with sepsis. However, most of these studies did not follow up to determine the long-term impacts on patients’ fluid and electrolyte balance after high-volume resuscitation strategies. This study sought to investigate the results of the aggressive fluid resuscitation measures used on patients with sepsis over the course of 7 days following resuscitation. An initial 3528 adult patients with sepsis who met inclusion criteria from the Medical Information Mart for Intensive Care IV database were collected. The total 7-day fluid balance of each patient was calculated and categorized into quartiles. Univariate Cox regression, lasso regression, backward stepwise elimination, and multivariate Cox regression were performed to search for variables related to survival during hospitalization. To determine the critical point of patients’ fluid balance over 7 days, a restricted cubic spline regression model with 4 knots was performed. In addition, an inverse probability of treatment weighting analysis was conducted to confirm our findings. The median 7-day fluid balance is 5321.4 (interquartile range, 848.5–10,404.0) mL. The observed 28-day mortality in this cohort was 21.6%. Both before and after the inverse probability of treatment weighting analysis, the first 7-day fluid balance in the intensive care unit was significantly related to mortality during hospitalization (*P* < .001). A restricted cubic spline regression analysis indicated when the 7-day fluid balance was equal to 5243.3ml, the heart rate value was ≈1. A 7-day fluid balance < 5243.3 mL was considered a protective factor, while a balance > 5243.3 mL was considered a risk factor for patient mortality. To be clear, this study does not advocate against aggressive fluid resuscitation in patients with sepsis. However, clinicians walk a fine line with the extent of the resuscitation volume given to patients with sepsis. As a result of this study, it is highly advised that fluid resuscitation in patients with sepsis be limited to ≈5200-mL 7-day fluid balance for optimal clinical benefit.

## 1. Introduction

Fluid management in patients with sepsis is a cornerstone for critical care. It is conventionally taught that aggressive fluid resuscitation is beneficial in patients with sepsis.^[1–3]^ Recent studies have come to question the “more is better” mentality in fluid administration for patients with sepsis, and early excessive liquid administration may cause an adverse prognosis.^[4]^ Some studies even suggested a negative correlation between liquid balance and prognosis.^[5,6]^ Therefore, a subsequent prospective study^[7]^ investigated whether conservative fluid resuscitation could improve patient survival and reduce complications. At the same time, fluid management strategies after resuscitation have gradually attracted attention. Research has found that after resuscitation, the potential benefits of liquid administration should be weighed against the risk of organ edema and the harmful effects of liquid components.^[8]^

Despite the studies listed above, the literature studying the impact of fluid balance after resuscitation on the short-term outcome of patients is rare. Our study hypothesized that overly aggressive fluid strategy in patients with sepsis can lead to adverse outcomes. To test this theory, the 7-day fluid balance of patients with sepsis in the intensive care unit (ICU) was examined retrospectively in a large database and correlated with prognosis.

## 2. Materials and methods

### 2.1. Study design

A retrospective cohort study was conducted herein. The Medical Information Mart for Intensive Care IV (MIMIC-IV; v1.0) database contains comprehensive and high-quality data of well-defined and characterized ICU patients admitted to ICUs at the Beth Israel Deaconess Medical Center between 2008 and 2019.^[9]^ A member of our team (W.S.) accessed the database for extraction (certification number 48845992).

### 2.2. Study population

**Figure 1.**
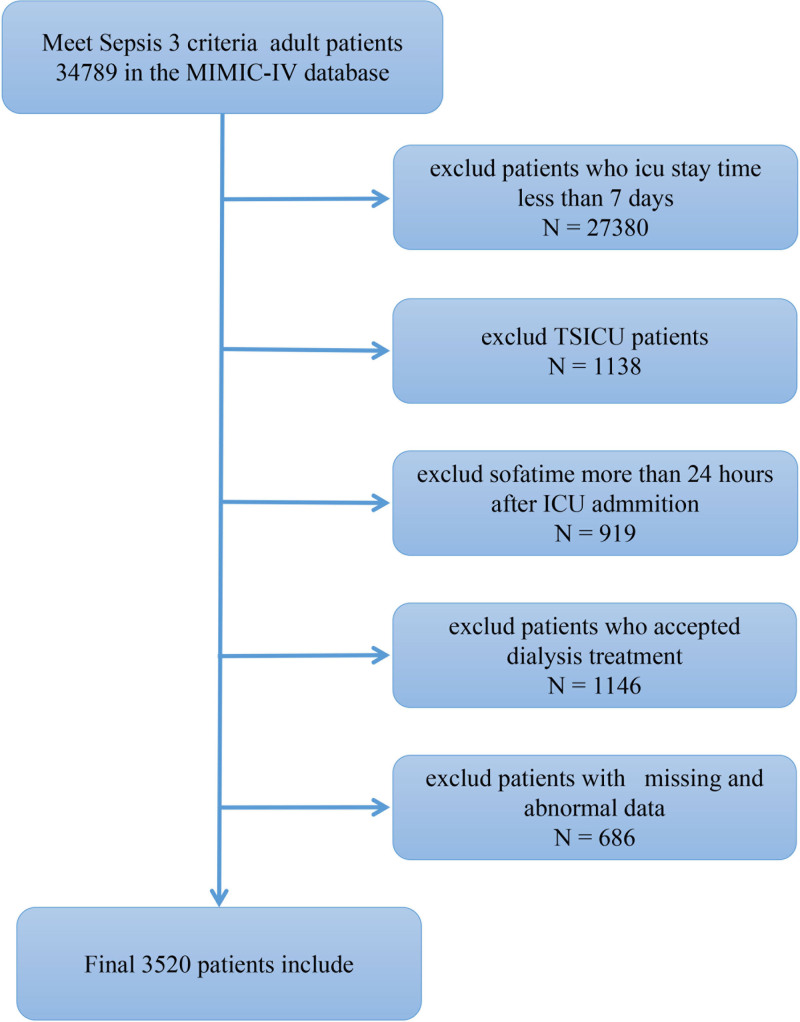
Study flowchart. ICU = intensive care unit, MIMIC-IV = Medical Information Mart for Intensive Care IV, TSICU = trauma and surgical intensive care unit.

A total of 34,789 adult patients with sepsis (defined by sepsis-3 criteria) in the MIMIC-IV 1.0 database were eligible for inclusion.^[10]^ Patients were excluded if they were in the ICU for <7 days, were admitted for trauma, had a sequential organ failure assessment score time collected over 24 hours before or after ICU admission, or underwent kidney transplantation within 7 days of ICU admission. This study only observed survival during hospital admission (Fig. 1).

### 2.3. Variable extraction

Patient data such as age, gender, weight, past medical history, sequential organ failure assessment score, complete blood count, basic metabolic panel, magnesium, and vital signs were collected. Any intervention with vasoactive drugs, invasive mechanical ventilation, presence of acute kidney injury, or septic shock within 7 days of ICU admission were collected.

### 2.4. Fluid calculation

The 7-day fluid balance was determined by subtracting the net fluid intake from the net fluid output. Specific fluids collected for output were urine, drain, and gastrointestinal output losses.

### 2.5. Statistical analysis

We categorized patients into quartiles by their 7-day fluid balance. Univariate Cox regression, lasso regression, backward stepwise elimination, and multivariate Cox regression were performed to screen variables related to survival during hospitalization. Baseline variables that showed a relationship with the outcome (*P* < .10) were included in the next analysis including age, weight, coronary disease, chronic kidney disease, septic shock, hemoglobin, blood urea nitrogen, creatinine, systolic blood pressure, mean temperature, and the minimum of oxygen saturation during the first 24 hours after ICU admission + 6 hours before ICU admission.

Inverse probability of treatment weighting was employed to adjust the covariates to validate our findings. To further identify the critical point of the 7-day fluid balance, a restricted cubic spline regression model with 4 knots was performed. All statistical analyses were performed using R statistical software (version 4.1.3), and *P* < .05 was considered statistically significant.

## 3. Results

**Figure 2.**
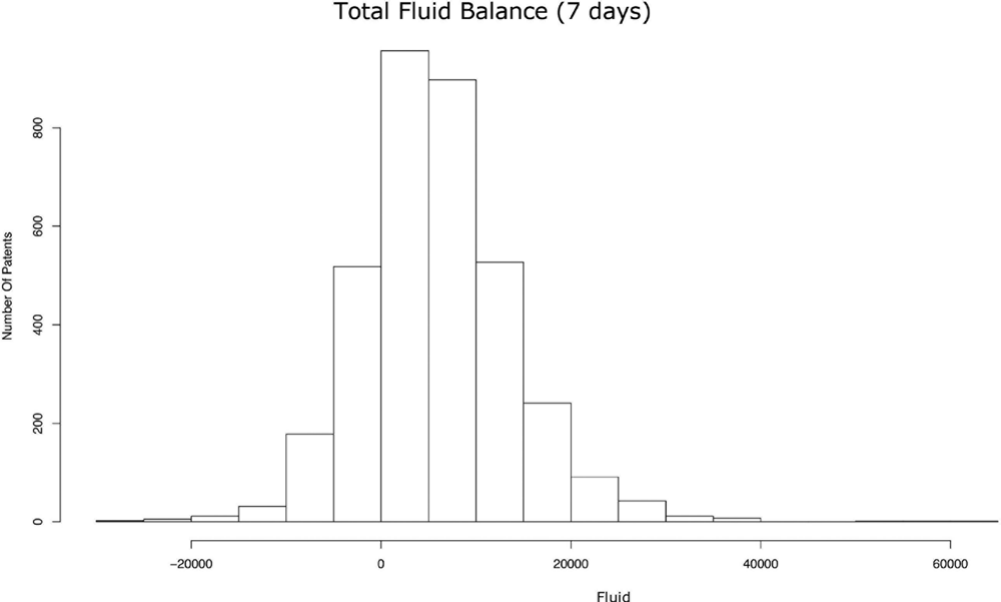
Distribution of patients’ 7-day fluid balance after intensive care unit admission in our cohort.

The MIMIC-IV database included 34,789 adult patients with sepsis, but only 3520 patients met the criteria for this study (Fig. 1). The median of the 7-day fluid balance was 5321.4 mL (interquartile range, 848.5–10,404.0 mL; Fig. 2). Characteristics of the patient population broken down by fluid volume are shown in Table 1. The observed 28-day mortality in our cohort was 21.6%.

**Table 1:** Cohort summary.

Covariates	First quartile (N = 880)	Second quartile (N = 880)	Third quartile (N = 880)	Fourth quartile (N = 880)	Among all patients (N = 3520)
7-d fluid balance (ml)	−3420 (3970)	3150 (1280)	7630 (1410)	16,100 (5600)	5860 (7930)
Age, yr	65.0 (15.4)	64.8 (15.9)	64.6 (15.3)	63.2 (15.5)	64.4 (15.5)
Male, %	511 (58.1)	482 (54.8)	494 (56.1)	532 (60.5)	2019 (57.4)
Weight, kg	89.8 (30.2)	81.3 (23.8)	80.0 (23.6)	81.5 (26.0)	83.2 (26.3)
SOFA score	3.56 (1.77)	3.40 (1.72)	3.40 (1.65)	3.87 (1.99)	3.56 (1.79)
AKI, %	793 (90.1)	768 (87.3)	786 (89.3)	828 (94.1)	3175 (90.2)
Septic shock, %	193 (21.9)	185 (21.0)	250 (28.4)	384 (43.6)	1012 (28.8)
Interventions					
Vasopressor use	529 (60.1)	529 (60.1)	556 (63.2)	676 (76.8)	2280 (64.8)
Invasive ventilation use	664 (75.5)	749 (85.1)	763 (86.7)	763 (86.7)	763 (86.7)
Comorbidities					
Hypertension, %	230 (26.1)	251 (28.5)	235 (26.7)	258 (29.3)	974 (27.7)
Diabetes, %	330 (37.5)	275 (31.3)	280 (31.8)	229 (26.0)	1114 (31.6)
Coronary, %	296 (33.6)	209 (23.8)	161 (18.3)	153 (17.4)	819 (23.3)
COPD, %	147 (16.7)	105 (11.9)	69 (7.8)	53 (6.0%)	374 (10.6)
Heart failure, %	511 (58.1)	288 (32.7)	225 (25.6)	211 (24.0)	1235 (35.1)
CKD, %	243 (27.6)	173 (19.7)	165 (18.8)	140 (15.9)	721 (20.5)
Stroke, %	93 (10.6)	155 (17.6)	216 (24.5)	121 (13.8)	585 (16.6)
Vital signs					
Heart_rate_min	72.5 (15.5)	72.2 (16.1)	72.9 (16.1)	78.2 (17.7)	74.0 (16.5)
Heart_rate_max	108 (21.3)	108 (21.8)	111 (21.4)	116 (22.7)	111 (22.0)
Heart_rate_mean	87.5 (15.9)	87.6 (16.7)	89.3 (16.6)	94.6 (18.0)	89.8 (17.0)
SBP_min	88.0 (16.1)	88.1 (16.7)	88.9 (16.7)	84.7 (16.7)	87.4 (16.6)
SBP_max	147 (23.1)	151 (24.7)	152 (25.3)	147 (25.0)	149 (24.6)
SBP_mean	115 (14.9)	116 (15.4)	118 (16.1)	112 (15.6)	115 (15.6)
DBP_min	45.0 (10.9)	44.9 (10.8)	45.3 (10.9)	44.0 (11.1)	44.8 (10.9)
DBP_max	88.8 (20.0)	88.5 (20.3)	90.9 (22.2)	87.7 (21.0)	89.0 (20.9)
DBP_mean	62.3 (10.2)	61.9 (10.4)	62.8 (10.5)	61.0 (9.92)	62.0 (10.3)
MBP_min	56.1 (14.0)	56.1 (14.0)	57.2 (13.3)	53.7 (14.8)	55.8 (14.1)
MBP_max	107 (28.2)	106 (23.5)	110 (29.6)	107 (28.7)	107 (27.6)
MBP_mean	77.0 (9.94)	77.0 (10.2)	78.4 (10.7)	75.6 (10.1)	77.0 (10.3)
Respiratory_rate_min	13.4 (4.05)	13.4 (4.07)	13.2 (3.96)	13.4 (4.01)	13.4 (4.02)
Respiratory_rate_max	29.9 (6.57)	29.7 (6.99)	30.0 (7.46)	30.6 (7.29)	30.1 (7.09)
Respiratory_rate_mean	20.7 (4.21)	20.7 (4.37)	20.6 (4.14)	21.2 (4.41)	20.8 (4.29)
Temperature_min	36.3 (0.753)	36.3 (1.06)	36.4 (0.813)	36.3 (0.842)	36.3 (0.874)
Temperature_max	37.5 (0.809)	37.7 (0.844)	37.8 (0.908)	37.8 (0.971)	37.7 (0.891)
Temperature_mean	36.9 (0.615)	37.0 (0.628)	37.1 (0.681)	37.0 (0.731)	37.0 (0.668)
SpO_2__min	89.7 (7.33)	90.9 (6.65)	91.6 (5.93)	90.9 (7.46)	90.8 (6.90)
SpO_2__max	99.4 (1.14)	99.6 (1.02)	99.6 (0.970)	99.7 (0.834)	99.6 (1.00)
SpO_2__mean	96.3 (2.47)	96.9 (2.18)	97.2 (2.14)	97.1 (2.09)	96.9 (2.25)
Laboratory tests					
Hemoglobin_min	9.92 (2.25)	10.1 (2.20)	9.98 (2.30)	9.58 (2.31)	9.89 (2.27)
Hemoglobin_max	11.1 (2.32)	11.4 (2.24)	11.4 (2.27)	11.4 (2.33)	11.4 (2.30)
Platelets_min	197 (105)	202 (114)	199 (115)	183 (121)	195 (114)
Platelets_max	233 (118)	243 (132)	245 (133)	240 (146)	240 (133)
WBC_min	11.3 (7.44)	11.2 (6.38)	11.2 (6.66)	11.5 (7.34)	11.3 (6.97)
WBC_max	14.7 (9.06)	15.5 (10.4)	15.7 (9.23)	16.7 (9.82)	15.6 (9.67)
BUN_min	30.5 (21.8)	26.7 (22.0)	24.3 (18.4)	27.6 (21.0)	27.3 (20.9)
BUN_max	35.1 (23.5)	31.1 (23.4)	29.2 (21.1)	33.1 (24.8)	32.1 (23.3)
Calcium_min	8.09 (0.799)	8.03 (0.902)	7.91 (0.834)	7.64 (0.950)	7.92 (0.890)
Calcium_max	8.59 (0.792)	8.58 (0.917)	8.54 (0.842)	8.50 (2.00)	8.55 (1.24)
Chloride_min	100 (6.73)	102 (6.67)	102 (6.65)	103 (7.68)	102 (7.02)
Chloride_max	104 (6.75)	106 (6.57)	107 (6.55)	108 (7.48)	106 (7.02)
Creatinine_min	1.27 (0.806)	1.12 (0.717)	1.06 (0.727)	1.16 (0.829)	1.16 (0.774)
Creatinine_max	1.50 (0.903)	1.35 (0.848)	1.30 (0.856)	1.47 (1.01)	1.41 (0.911)
Glucose_min	124 (43.3)	123 (44.1)	122 (41.4)	119 (42.2)	122 (42.8)
Glucose_max	189 (117)	177 (90.1)	185 (108)	200 (223)	188 (144)
Sodium_min	137 (5.29)	137 (5.52)	137 (5.65)	137 (6.59)	137 (5.79)
Sodium_max	140 (4.98)	141 (5.18)	141 (5.61)	141 (6.70)	141 (5.67)
Potassium_min	3.87 (0.587)	3.88 (0.583)	3.78 (0.571)	3.78 (0.586)	3.83 (0.584)
Potassium_max	4.67 (0.873)	4.60 (0.836)	4.53 (0.840)	4.64 (0.861)	4.61 (0.854)
Phosphate_min	3.42 (1.20)	3.27 (1.10)	3.05 (1.07)	3.17 (1.23)	3.22 (1.16)
Phosphate_max	4.18 (1.39)	4.05 (1.67)	3.82 (1.26)	4.17 (1.48)	4.05 (1.47)
Magnesium_min	1.94 (0.375)	1.89 (0.382)	1.81 (0.350)	1.77 (0.353)	1.85 (0.371)
Magnesium_max	2.27 (0.512)	2.27 (1.06)	2.25 (1.34)	2.19 (0.407)	2.24 (0.914)

For all continuous covariates, the mean values and standard deviations are reported.

AKI = acute kidney injury, BUN = blood urea nitrogen, CKD = chronic kidney disease, COPD = chronic obstructive pulmonary disease, DBP = diastolic blood pressure, MBP = mean blood pressure, SBP = systolic blood pressure, SOFA = sequential organ failure assessment, SpO_2_ = oxygen saturation, WBC = white blood cell.

**Figure 3.**
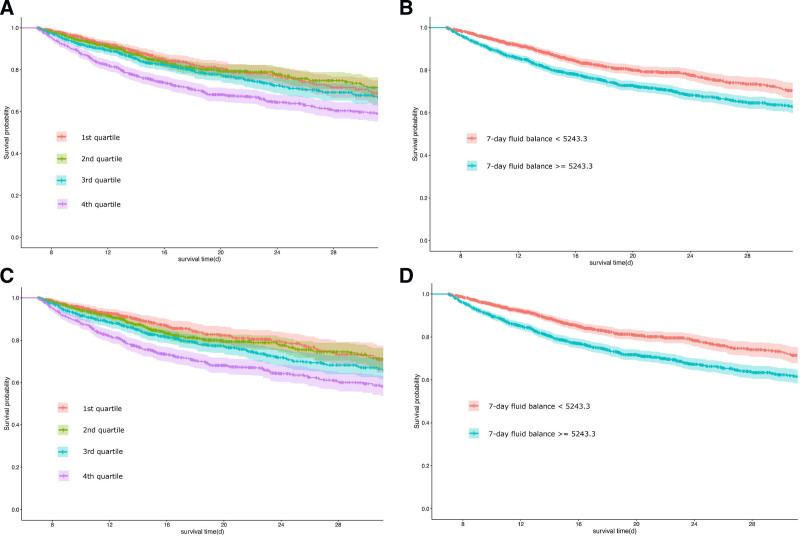
The Kaplan-Meier survival curve of the 2 and 4 groups. (A and C) Patients were divided into 4 quantiles of fluid balance. (B and D) Patients were divided into 2 groups according to the restricted cubic spline regression. These results show a significant correlation between the 7-day fluid balance and overall survival after 28 days regardless of the inverse probability of treatment weighting analysis.

After dividing patients into quartiles based on the volume of the 7-day fluid balance, a significant correlation with hospital survival was observed. In addition, these correlations were significant after the inverse probability of treatment weighting analysis (*P* < .001; Fig. 3A and 3C). The results of the restricted cubic spline regression showed similar results (*P* < .001; Fig. 3B and 3D).

**Figure 4.**
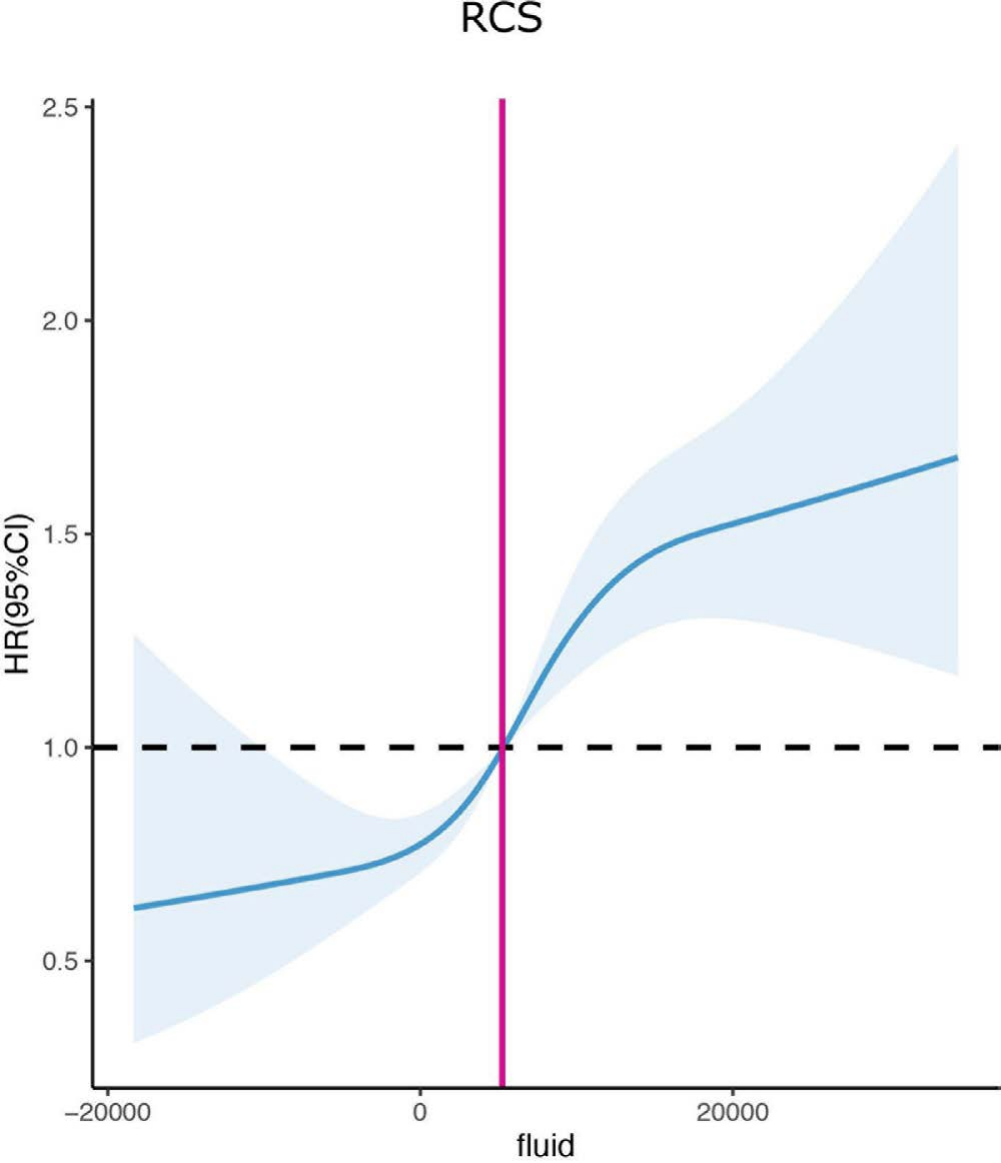
Restricted cubic splines for the association between 7-day fluid balance and survival rate during hospitalization. When the 7-day fluid balance was equal to 5243.3 mL, the HR value was ≈1. HR = heart rate.

After a restricted cubic spline regression analysis, it was found that the 7-day fluid balance was equal to 5243.3 mL when the heart rate value was ≈1. As a result, a 7-day fluid balance of <5243.3 and >5243.3 mL was correlated with a better and worse prognosis, respectively (Fig. 4)

## 4. Discussion

The fluid balance of patients with sepsis within the first 7 days of ICU admission was compared to the overall outcome during hospitalization. The results from this study confirm the hypothesis and suggest that a fluid balance of < 5240 mL was mutually beneficial to both acute and short-term patient outcomes. However, resuscitation volumes leading to a fluid balance of over 5240 mL were associated with a higher rate of mortality during hospitalization.

The original guidelines of early goal-directed therapy (EGDT) research published over a decade ago encouraged the use of aggressive fluid resuscitation.^[1]^ especially crystalloids, resulting in excessive use. However, modern studies have begun to shed light on the optimal point of fluid balance for patients with sepsis.^[4]^ The results obtained herein provide additional evidence to suggest that more is not always better, and the optimal point of fluid balance for patients with sepsis ranged around 5240 mL.

From a biological standpoint, the rapid release of inflammatory factors early in the pathophysiology of sepsis can greatly affect vasculature resistance, permeability, capacity, and arterial dilation. The overall result of these changes can cause decreased plasma colloid osmotic pressure, insufficient arterial filling, microcirculation dysfunction, and secondary interstitial edema, with insufficient systemic perfusion and local tissue ischemia.^[11–13]^

Traditional methods regarding fluid resuscitation in patients with sepsis suggest restoring the intravascular volume of cardiac output and oxygen delivery.^[14]^ Specifically, crystalloids are often used to increase preload^[15]^ without consideration of their effects on systemic organs.^[16]^ In addition, septic myocardial damage during fluid infusion is often unintentionally ignored or underestimated^[17]^ as an expected complication of aggressive therapy. If a large amount of fluid is retained, excess fluid can leak out of capillaries and aggravate interstitial edema.^[18]^ Recent studies have begun to explore the accuracy of early liquid management,^[19,20]^ and the ProCESS trial (Protocolized Care for Early Septic Shock) compared the initial EGDT methods,^[21]^ improved EGDT, and conventional treatment. While no significant difference in mortality was observed, the mortality of the improved EGDT group was significantly less compared to the initial EGDT study (21% vs 44%). This difference may arise from the lower volume of fluid resuscitation in 72 hours (7.2 vs 13.4 L). Therefore, it is speculated that the difference in mortality may be partly attributed to the difference in fluid resuscitation strategies.

A patients’ fluid balance is a dynamic equilibrium and is affected by a variety of factors.^[22–25]^ Studies using methods to dynamically observe the effect of liquid administration throughout the course of the disease have revealed the incidence of acute kidney injury and injury to other organs as a result of aggressive fluid administration.^[26,27]^ In this study, 1000 patients with acute lung injury were randomly divided into the restricted fluid treatment group and the free fluid treatment group, all of whom were treated with a clear treatment plan for 7 days. In the restricted liquid therapy group, the pulmonary function was significantly improved, and the duration of mechanical ventilation and intensive care was shorter than in the free fluid treatment group. In contrast, 2 recent studies on restrictive fluid management after septic resuscitation achieved a cumulative fluid balance difference of approximately 1 liter between groups within 5 days,^[28]^ so there was no difference in clinical results. Therefore, a “less is more” strategy may be more beneficial for fluid resuscitation of septic shock.

Some studies have divided liquid resuscitation into 4 stages: the salvage stage, the optimization stage, the stabilization stage, and the de-escalation stages.^[29]^ However, there are no clear guidelines dividing the 4 stages. In emergent situations, it is critical to ensure a minimum blood pressure to support end-organ perfusion. As such, administration of high volumes of crystalloid is clinically necessary. Yet, the duration of these critical measures should be scrutinized to provide the best overall outcome throughout the course of hospitalization. After systemic circulation is restored and the administration of antibiotics, it is important to allow the patient to rebound to a state of equilibrium. This study provides evidence to suggest that the optimal 7-day fluid balance for patients should be <5240 mL for the best outcomes. Future studies along this direction should focus on determining the difference between crystalloid and colloid fluid treatment regarding outcome, as well as the dose of vasoactive drugs.

The importance of fluid balance should not be overlooked. Fluid overload in patients with sepsis may cause more harm than good in the long term due to damage to systemic organs.

We would like to express caution that our conclusions regarding liquid management may differ from current guidelines, as we have not subdivided the types of liquids. In addition, the liquid management time that we observed was longer, and there were no restrictions on the timing of initial liquid resuscitation or initiation of pressure-boosting drugs. Therefore, although our results certainly demonstrate the benefits of limiting strategies during the initial and liquid optimization stages, they do not directly point to specific guidelines.

## 5. Conclusion

The net fluid balance in patients with sepsis 7 days following ICU administration was significantly related to mortality during hospitalization. Specifically, a 7-day fluid balance > 5240 mL was associated with poor outcomes. Future multicenter retrospective studies, prospective studies, and randomized controlled trials are needed to further clarify the appropriate range of fluid balance within the first 7 days and to further refine the management of septic resuscitation.

## Acknowledgments

The authors would like to thank the Massachusetts Institute of Technology and the Beth Israel Deaconess Medical Center for the Medical Information Mart for Intensive Care project.

## Author contributions

**Software:** Wenwen Song.

**Writing – original draft:** Zheng Yang, Wenwen Song.

**Writing – review & editing:** Zheng Yang, Zhanli Shi, Wenwen Song.

**Funding acquisition:** Zheng Yang, Zhanli Shi.

**Methodology:** Zheng Yang, Zhanli Shi.
